# Virtual reality-based interventions targeting sports injury-related risk factors: a systematic review of biomechanical, neuromuscular, and functional outcomes

**DOI:** 10.3389/fspor.2026.1744369

**Published:** 2026-06-30

**Authors:** Gholam Rasul Mohammad Rahimi, Nasser Mohammad Rahimi, Matthias Wilhelm Hoppe

**Affiliations:** 1 Department of Exercise Physiology, Ayandegan-e-Sharq Healthcare Center, Mashhad, Iran; 2 Department of Sports Injuries and Corrective Exercises, Ayandegan-e-Sharq Healthcare Center, Mashhad, Iran; 3 Department of Exercise Science, Institute of Sport Science and Motology, Philipps University of Marburg, Marburg, Germany

**Keywords:** balance training, digital intervention, injury-related risk factors, sports performance, virtual reality

## Abstract

**Background:**

Virtual reality (VR)-based interventions are increasingly applied in sports training and musculoskeletal rehabilitation. However, their potential role in modifying lower-extremity injury-related risk factors in athletic populations remains incompletely understood.

**Methods:**

Following PRISMA guidelines, PubMed, Scopus, Web of Science, and SportDiscus were searched up to September 25th, 2025. Eligible studies included randomized, controlled, quasi-experimental, or within-subject experimental designs evaluating immersive, semi-immersive, or non-immersive VR interventions in junior to young-adult athletes. Comparator conditions included conventional training, alternative exercise interventions, no-intervention controls, placebo conditions, or non-VR comparisons. Outcomes addressed biomechanical, neuromuscular, functional, perceptual-cognitive, or psychological factors potentially relevant to sports injury risk. Methodological quality was assessed using the Downs and Black checklist.

**Results:**

Thirty studies met the inclusion criteria. Interventions ranged from single-session biofeedback exposure to 4–16-week neuromuscular, balance, perceptual-cognitive, or sensorimotor programs across multiple sports. Most studies reported improvements in at least one biomechanical, neuromuscular, functional, perceptual-cognitive, or psychological outcome; particularly, in lower-extremity movement control, balance, coordination, and reaction efficiency. However, several acute studies demonstrated transiently less favorable movement mechanics during highly immersive or cognitively demanding tasks. Importantly, no included study evaluated injury incidence as a primary preventive outcome. Downs and Black scores ranged from 14 to 26 (mean = 19.6 ± 3.1), indicating overall fair-to-good methodological quality.

**Discussion:**

The evidence suggests that VR-based training can effectively modify several surrogate lower-extremity injury-related risk factors; particularly, those associated with biomechanics and neuromuscular control. Nevertheless, the lack of longitudinal data and the scarcity of injury-incidence outcomes limit conclusions regarding real-world preventive efficacy.

**Conclusions:**

VR-based interventions may represent a promising adjunct to conventional neuromuscular and perceptual-cognitive training approaches for modifying surrogate lower-extremity injury-related factors. Nevertheless, substantial heterogeneity, limited longitudinal follow-up, and the absence of injury-incidence outcomes restrict conclusions regarding definitive preventive efficacy. Future adequately powered randomized trials with standardized protocols and verified injury outcomes are required.

**Systematic Review Registration:**

https://www.crd.york.ac.uk/PROSPERO/view/CRD420251163302, identifier CRD420251163302.

## 1 Introduction

Sports participation, while offering substantial health and developmental benefits, is inherently associated with an injury risk across all ages, sexes, and competition levels (1). Musculoskeletal injuries of the lower extremity—ranging from ankle sprains and hamstring strains or tendinopathies to more severe anterior cruciate ligament (ACL) ruptures—impose significant burdens on athletes through pain, time loss, and long-term consequences such as post-traumatic osteoarthritis (2, 3). The economic impact is also profound, with ACL injuries alone costing professional athletes and leagues hundreds of millions of dollars in lost earnings and high individual economic losses (4–6). Consequently, multidimensional injury-prevention strategies are critical for safeguarding athlete health, sustaining performance, and promoting career longevity.

Neuromuscular training programs, integrating elements like plyometric, balance, and agility exercises, have consistently demonstrated their efficacy in reducing injury risk by enhancing dynamic joint stability, improving movement control, and optimizing landing mechanics (7, 8). However, their translation into real-world settings remains limited due to low adherence and motivational barriers; particularly, among youth and competitive athletes (9, 10). Monotony, lack of engaging feedback, and insufficient sport specificity often diminish compliance and long-term program maintenance, thereby reducing preventive effectiveness. In addition, the implementation of conventional injury-prevention programs in real-world settings may be limited by accessibility barriers, logistical demands, and reduced adaptability to individualized or remote training environments (11, 12). In this context, younger and adolescent athletes represent an especially relevant population for digital and virtual training approaches, given their superior digital literacy, adaptability, and intrinsic motivation to engage with technology-based learning environments. These characteristics may make them particularly responsive to virtual reality (VR) training (13).

VR has emerged as a promising approach to addressing these limitations by combining engagement, real-time feedback, and sport-specific contextualization, and the potential for accessible, scalable, and remotely deliverable training environments within an interactive digital setting (14, 15). VR systems enable athletes to interact within gamified, three-dimensional simulations that reproduce sport-specific contexts and provide real-time visual and auditory feedback, fostering intrinsic motivation and motor learning (16, 17). Beyond rehabilitation, VR is increasingly applied in athletic settings for the modification of injury-related risk factors and performance-oriented adaptations, targeting improved neuromuscular control, decision-making, and biomechanical efficiency in realistic, feedback-rich contexts. Controlled experiments show that VR can elicit ecologically valid, sport-specific biomechanical responses and facilitate the identification of high-risk movement patterns such as excessive knee valgus or trunk displacement (18–20).

VR-based training has also produced measurable improvements in factors commonly associated with sports-injury risk, including balance, proprioception, and lower-limb coordination (21–24). Moreover, VR-augmented biofeedback protocols have reduced peak knee abduction moments and enhanced landing strategies—variables frequently linked to ACL injury mechanisms (19, 25, 26). In addition to biomechanical and neuromuscular adaptations, immersive environments can improve cognitive-motor integration, enhancing reaction time, decision-making, and attentional control under pressure—capacities considered relevant to real-time movement regulation and sport performance (27–29). Nevertheless, the extent to which improvements in these surrogate biomechanical or neuromuscular markers translate into actual reductions in injury incidence remains incompletely understood. Contemporary injury-prevention literature has increasingly questioned the predictive and causal validity of several commonly used risk markers, emphasizing that associations with injury risk may be multifactorial, context-dependent, and not necessarily causal (30). Therefore, improvements in such parameters should be interpreted cautiously and not assumed to directly indicate injury reduction.

Despite growing evidence, a comprehensive synthesis of VR-based interventions targeting biomechanical, neuromuscular, functional, perceptual-cognitive, and psychological factors associated with sports-injury risk remains limited. Prior reviews have predominantly combined preventive and rehabilitative studies (14, 15) or focused narrowly on ACL rehabilitation (17), while often failing to critically distinguish between modifications in injury-related surrogate outcomes and verified reductions in injury occurrence. This distinction is particularly important given the increasingly recognized complexity and multifactorial nature of sports-injury mechanisms, in which isolated biomechanical or neuromuscular risk markers may not directly predict injury incidence (30). Therefore, the current systematic review aims to critically appraise and synthesize empirical evidence on the effectiveness of VR-based interventions in modifying biomechanical, neuromuscular, functional, and psychological risk factors predominantly related to lower-extremity sports injuries. Importantly, the review focuses primarily on injury-related surrogate outcomes rather than confirmed injury-incidence reductions. The overarching goal is to inform the development of evidence-based, scalable digital frameworks for proactive injury-risk management and performance enhancement in athletic populations across varying performance levels.

## 2 Materials and methods

This systematic review was conducted in accordance with the Preferred Reporting Items for Systematic Reviews and Meta-Analyses (PRISMA) guidelines (31). The review protocol was prospectively registered with the PROSPERO database (Registration Number: CRD420251163302).

### 2.1 Search strategy and data sources

A comprehensive systematic search was conducted across four electronic databases from their inception up to September 25th, 2025: PubMed/MEDLINE, Scopus, Web of Science, and SportDiscus (EBSCOhost). The search strategy combined keywords and controlled vocabulary (e.g., MeSH terms) across three core concepts: (1) the intervention (VR and related technologies), (2) the problem (sports injuries and injury prevention), and (3) the population (athletes or physically active individuals participating in organized sports or structured training). To ensure a comprehensive review, the reference lists and citations of all included studies and relevant systematic reviews were also manually screened. The full, detailed search strategy for each database is provided in the Supplementary Table S1.

### 2.2 Study selection process

**Figure 1:**
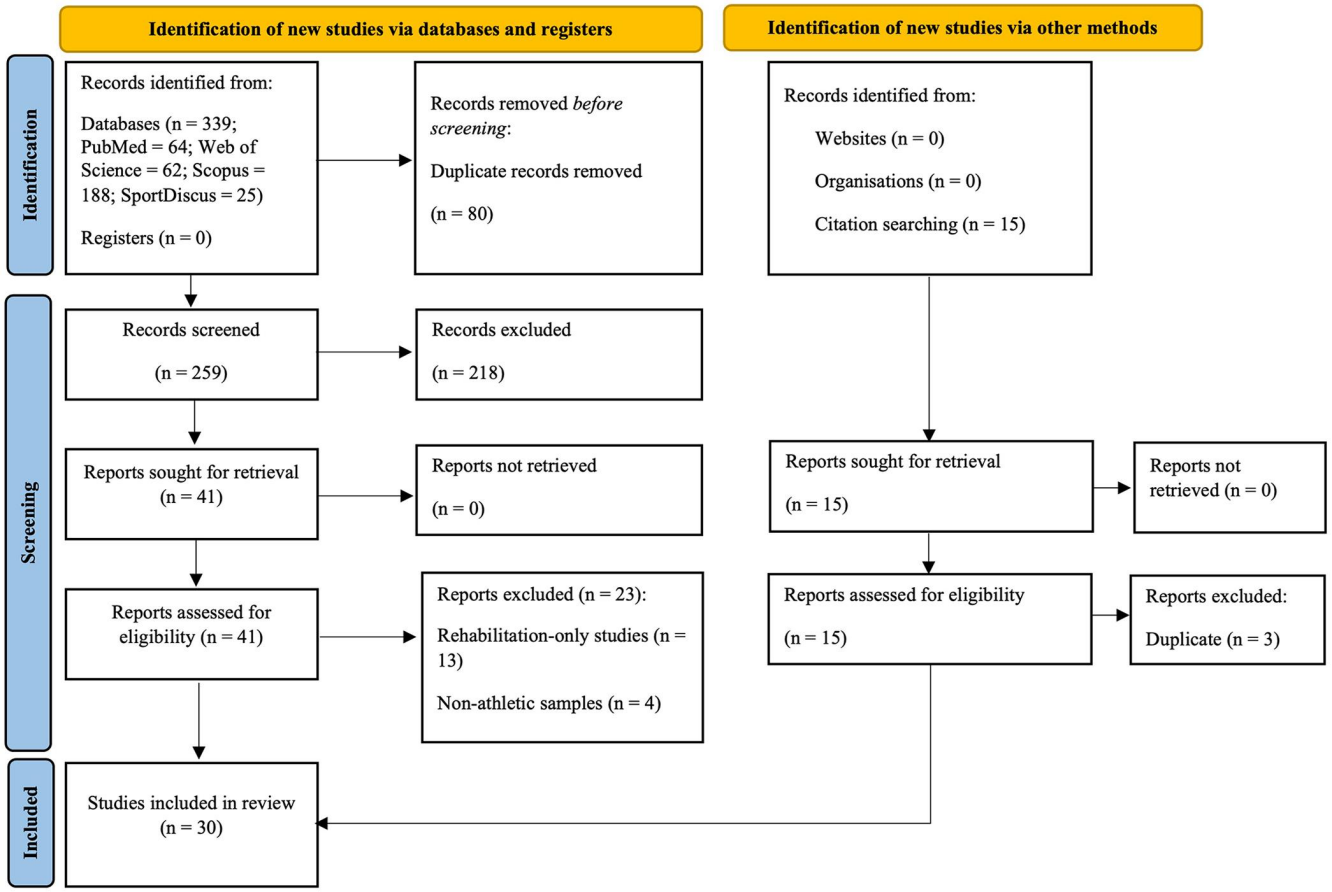
PRISMA (Preferred Reporting Items for Systematic Reviews and Meta-Analyses) flow diagram of study selection process.

After importing all identified records into EndNote for deduplication, titles and abstracts were independently screened by two reviewers against the eligibility criteria. All methodological procedures, including study screening and selection, were completed independently by two researchers. When disagreement arose, consensus was reached through discussion. Full-text articles of potentially eligible studies were retrieved and independently assessed. Reasons for exclusion were noted, and the process was visualized in a PRISMA flow diagram (Figure 1).

### 2.3 Eligibility criteria

The eligibility criteria were developed according to the PICOS framework (Population, Intervention, Comparator, Outcomes, Study Design):

*Population (P):* Junior, adolescent, or young adult athletes participating in any sport. Studies including participants with specific musculoskeletal complains (e.g., functional ankle instability, chronic low back pain) were also included if the intervention focused on the modification of injury-related risk factors, secondary prevention, or performance-related adaptations rather than post-injury rehabilitation or clinical recovery as the primary objective. However, studies were excluded if participants were undergoing post-injury rehabilitation or medical treatment as the primary objective, or if non-athletic populations, such as patients with neurological, cognitive, or unrelated chronic diseases were involved. The term “young adult” in this review refers to individuals aged approximately 18–35 years; studies exclusively enrolling participants over 40 years were not identified and would have been excluded due to the different biomechanical and neuromuscular profiles as well as assumed less digital literacy of older adults.

*Intervention (I)*: VR-based interventions designed for sports injury prevention-particularly of the lower extremity-or for performance-related adaptations associated with neuromuscular control, perceptual-cognitive function, balance, coordination, or movement quality, including immersive (head-mounted displays), semi-immersive (projector-based), or non-immersive (screen-based or biofeedback) VR platforms.

*Comparator (C)*: Traditional injury-prevention programs (e.g., conventional neuromuscular training), alternative exercise forms (e.g., isokinetic or balance training), no-intervention controls, or within-subject designs comparing VR to non-VR conditions.

*Outcomes (O)*: Studies reporting objective measures related to sports injury risk factors, primarily at the lower extremity, including: biomechanical parameters (e.g., joint angles, ground reaction forces, knee abduction moment), neuromuscular outcomes (e.g., EMG activity, postural control, muscle activation timing), functional outcomes (e.g., balance performance, agility), and psychological outcomes (e.g., stress resilience, confidence, motivation). As the majority of studies examined surrogate risk-factor outcomes rather than injury incidence, this review focused on the modification of these predictors of injury risk. Accordingly, improvements in these variables were interpreted as potential indicators of injury-risk modification rather than direct evidence of injury reduction.

*Study design (S)*: Peer-reviewed empirical studies published in English. Randomized controlled, non-randomized controlled, within-subject comparative, and pre–post pilot designs were eligible. Case studies, editorials, conference abstracts, and non-empirical papers were excluded.

### 2.4 Data extraction

Data were extracted by two independent authors using a standardized, pre-piloted form structured according to the PICOS schema. Extracted data included: study design, sample characteristics (age, sport, performance level, and any relevant clinical history such as functional ankle instability), intervention details (type of VR, duration, and frequency), comparator details, outcome measures, and key findings. Discrepancies were resolved through discussion and consensus.

Performance level was classified retrospectively for each study according to the six-tier framework proposed by McKay et al. (2021), which distinguishes participants as Tier 0 (sedentary), Tier 1 (recreationally active), Tier 2 (trained/developmental), Tier 3 (highly trained/national), Tier 4 (elite/international), and Tier 5 (world class). This classification provided a standardized reference to compare study populations and to examine whether VR-related effects varied across competitive levels. However, because this classification was applied retrospectively based on the information reported in the included studies, some degree of subjectivity in participant categorization cannot be excluded.

### 2.5 Quality assessment

The methodological quality of the included studies was assessed using a modified version of the Downs and Black checklist (32), a validated tool designed to evaluate both randomized and nonrandomized healthcare intervention studies. The checklist consists of 27 items covering five domains: reporting (10 items), external validity (3 items), internal validity for bias (7 items), internal validity for confounding (6 items), and statistical power (1 item). Twenty-six items were rated dichotomously (yes = 1, no/unable to determine = 0), while one reporting item was scored on a 3-point scale (0–2), yielding a maximum possible score of 28. Higher scores indicate superior methodological quality. For interpretability, studies were categorized as excellent (26–28), good (20–25), fair (15–19), or poor (≤14) (33). Two reviewers independently conducted the quality assessments, with disagreements resolved through discussion. Importantly, quality classifications should be interpreted cautiously, as several studies categorized as “good” still demonstrated important methodological limitations, including small sample sizes, limited blinding procedures, and the absence of formal power calculations.

## 3 Results

### 3.1 Study selection

The database searches across PubMed/MEDLINE, Scopus, Web of Science, and SportDiscus yielded a total of 339 records. After removing 80 duplicates, 259 records were screened based on titles and abstracts. Of these, 218 were excluded as they did not meet the eligibility criteria. The full texts of 41 reports were retrieved and assessed for eligibility, and 23 were subsequently excluded (Supplementary Table S2); primarily, due to being rehabilitation studies only (*n* = 13), non-athletic samples (*n* = 4), non-empirical papers (*n* = 3), or reviews (*n* = 3). In addition, 15 records were identified through citation searching, 12 of which were included and 3 were duplicates of already retrieved articles. Ultimately, 30 studies (18, 19, 22–29, 34–52) met the inclusion criteria and were included in the final synthesis. Figure 1 illustrates the PRISMA flow of information through the identification, screening, and inclusion phases of the review.

### 3.2 Description of included studies

A total of 30 studies met the inclusion criteria and were incorporated in this review (Table 1). Collectively, the included studies involved 1,288 participants, ranging from adolescent to adult athletes across diverse sports, including soccer, basketball, handball, badminton, karate, wrestling, tennis, and other general or multi-sport training contexts. The studies were conducted in 14 countries, most frequently in the United States (*n* = 11), followed by Indonesia (*n* = 4), Iran (*n* = 3), Egypt (*n* = 2), and one each from Brazil, Turkey, the United Kingdom, Saudi Arabia, Germany, Belgium, Hungary, China, Croatia, and Tunisia. Publication years ranged from 2011 to 2025.

**Table 1:** Characteristics of included studies.

Author (Year)	Study design	Participants	Intervention details	Comparator details	Outcome measures	Key findings
Randomized controlled trials						
Faghihi & Khanmohammadi (2024) (51); Iran	RCT	Total: 32 male soccer players with chronic ankle instability Age: 22.4 ± 3.3 years Level: Tier 2	Type: Semi-immersive VR (Wii Balance Board) games: Single Leg Extension, Torso Twist, Soccer Heading, Table Tilt, Snowboard Slalom, etc. Frequency: 3 sessions/week for 4 weeks (12 sessions). Duration: 60 min each. Focus: enhancing anticipatory and compensatory postural strategies during ball kicking.	Conventional balance training (single-leg stance and hopping drills on stable/unstable surfaces; matched duration and frequency).	Primary: EMG (amplitude and onset latency of PL, TA, SOL, RF, BF, GM) during anticipatory (APA) and compensatory (CPA1–CPA2) phases of ball kicking. Secondary: Y-Balance Test (anterior, PM, PL directions); Cumberland Ankle Instability Tool (CAIT).	VR group: ↓ SOL activity in CPA2 (*p* = 0.033), earlier BF activation (*p* = 0.043); Balance group: ↑ GM activity in APA (*p* = 0.037), ↓ RF (*p* = 0.048), ↓ PL in CPA1–CPA2 (*p* ≤ 0.05). Both groups improved Y-balance (*p* = 0.020, Balance *p* = 0.032; PL: both *p* < 0.001) and CAIT scores (VR *p* = 0.002, Balance *p* < 0.001). No between-group differences. Both interventions enhanced neuromuscular timing and dynamic stability equally.
Fendrian et al. (2024) (48); Indonesia	RCT	Total: 30 male karate athletes Age: 14.0 ± 0.7 years Level: Tier 2	Virtual reality–based physical activity combining brain jogging, neurotracker, and aerobic circuit via immersive VR headset. Frequency: 3 sessions/week for 4 weeks (12 sessions). Duration per session: ∼45 min (5 min warm-up, 35 min VR, 5 min cool-down).	Regular physical activity (e.g., push-ups, running) for equal time and frequency.	Mental health: Depression, Anxiety, Stress (DASS-21). Cognitive function: Stroop color–word test (attention reaction time).	VR group: ↓ depression (*p* = 0.021); ↓ anxiety (*p* = 0.036); ↓ stress (*p* = 0.013); ↑ cognitive attention time (*p* < 0.001) compared with the control group. Qualitative feedback indicated that the VR activity was fun, motivating, and easy to use but limited by equipment availability.
Fortes et al. (2021) (29): Brazil	RCT	Total: 26 young soccer players Age: 14.2 ± 0.4 years Sport: Soccer Level: Tier 2	Type: Immersive VR (HMD) Content: Training focused on perceptual-cognitive skills (e.g., passing decision-making, visual search). Duration: 8 weeks	VSS: The same content was presented on a 2D screen.	Passing decision-making accuracy, visual search behavior, inhibitory control.	VR group: ↑ Passing decision-making accuracy vs. VSS group (*p* < 0.01). The VR environment proved more effective for enhancing perceptual–cognitive skills.
Marshall et al. (2023) (28); United Kingdom	RCT	Total: 36 soccer players Age: 28.7 ± 5.9 years Level: Tier 1	Type: Immersive VR (HMD) Content: Training soccer heading without physical impact Duration: 3 sessions over 7–10 days	Control group: No training in soccer heading	Real-world heading performance (goals scored and accuracy), perceived confidence, and self-efficacy.	VR group: ↑ Real-world heading accuracy (*p* = 0.042) and ↑ confidence (*p* < 0.001) vs. control group.
Mohammadi et al. (2023; 1) (23); Iran	Matched RCT	Total: 54 basketball players with FAI Age: 19–25 years Level: Tier 2	Type: Non-immersive VR (Wii exercises) Content: 12 sessions of Wii exercises Duration: 12 sessions, 3 days/week	Control group: 12 sessions of traditional training (balance exercises)	Subjective-sense of instability (CAIT score) and balance (SEBT).	VR group: ↑ Subjective sense of stability (*p* < 0.001) and ↑ balance performance (*p* < 0.05) vs. traditional training group.
Mohammadi et al. (2023; 2) (45); Iran	Matched RCT	Total: 50 male basketball players with and without functional ankle instability (FAI) Age: 21.9 ± 2.1 years Level: Tier 2	Non-immersive VR training using Wii Fit Plus balance and strength games (e.g., soccer heading, ski slalom, tight-rope walk, table tilt, rowing squat, sideways leg lift). Frequency: 3 sessions per week for 4 weeks (12 sessions). Duration per session: ≈45 min (5 min warm-up + 35 min VR + 5 min cool-down).	Control group: athletes without FAI who received no intervention.	Neurocognitive function: Simple reaction time (SRT), choice reaction time (CRT), and error rate using the Deary-Liewald Reaction Time Task (DLRT).	VR group: ↑ SRT and ↑ CRT (both *p* < 0.001); ↓ Error rate (*p* < 0.001). No significant changes in controls (*p* > 0.05). VR training enhanced information-processing speed and accuracy in athletes with FAI.
Mohamed & EI-Bedewy (2023) (46); Egypt	Experimental controlled trial	Total: 19 male junior wrestlers Age: 15–17 years Level: Tier 3	Non-immersive VR training using VR BOX glasses displaying 3-D recorded exercise videos via smartphones. Duration: 8 weeks. Frequency: 3 sessions per week (24 sessions). Session length: ≈90 min (20 min viewing + 70 min execution). Exercises targeted strength, power, flexibility and agility.	Conventional wrestling training program of equal duration and frequency without VR viewing.	Biomechanical & functional factors: Back and leg muscle strength (N·m via dynamometer); leg/arm power (broad jump, medicine-ball throw); strength-speed (bridge time ×3); flexibility (spinal/shoulder Dome test); agility (circle crawl test).	VR group: within-group: ↑ all performance abilities (*p* = 0.01). Between-group (post-test): VR group superior to control for all variables (*p* < 0.05), with the greatest gain in ↑ arm power. VR training substantially enhanced strength, power, flexibility, and agility in junior wrestlers vs. traditional training.
Novak et al. (2023) (44); Croatia	RCT	Total: 58 college tennis players Age: 22.9 ± 3.1 years Level: Tier 2	Immersive VR (ImproVR GmbH, Munich) program incorporating neuro-athletic, coordinative, and cognitive tasks for reflexive stability. Duration: single 5-min session. Frequency: one session only. Exercises included peripheral perception, gaze stability, and horizontal/diagonal saccades.	Placebo VR: watched a moving dot for 5 min (no reactive task).	Functional/neuromuscular: Dynamic balance via Y Balance Test (YBT) — anterior (ANT), posteromedial (PM), and posterolateral (PL) reach (left and right leg).	VR group: ↑ dynamic balance vs. control across multiple directions (ANT-L, PM-L, PM-R, PL-R; *p* ≤ 0.05). Right-handed group: ↑ ANT-R (*p* = 0.00), ↑ PM-R (*p* = 0.03); Left-handed group: ↑ ANT-L (*p* = 0.00), ↑ PM-L (*p* = 0.00), ↑ PL-L (*p* = 0.00), ↑ PM-R (*p* = 0.00), ↑ PL-R (*p* = 0.00). No significant changes in control group. VR training produced short-term improvements in dynamic balance.
Rahmadani et al. (2024) (43); Indonesia	RCT	Total: 20 basketball players (10 male, 10 female) Age: 18 ± 0.5 years Level: Tier 2	Immersive VR training using Meta Quest head-mounted display integrated with machine-learning adaptive feedback. Frequency: 3 sessions per week for 4 weeks (12 sessions total). Duration per session: 60 min (10 min warm-up + 40 min VR drills + 10 min performance feedback). Tasks: dribbling, passing, shooting under dynamic game-like conditions.	Conventional basketball training of same frequency and duration without VR or ML components. Executive	Function (EF-Attention) via Stroop test; Decision-Making (DM-Shooting, Passing, Dribbling) via French & Thomas game-based observation instrument.	VR + ML group: ↑ EF-attention (*p* < 0.001); ↑ DM-shooting (*p* < 0.001); ↑ DM-passing (*p* = 0.033); ↑ DM-dribbling (*p* < 0.001) vs. control. VR + ML training significantly enhanced attention and decision-making abilities in basketball athletes.
Nambi et al. (2020) (38); Saudi Arabia	RCT	Total: 40 university football players with chronic low back pain Age: 20.7 ± 1.1 years Level: Tier 2	Type: Non-immersive VR training Content: VR balance training. Duration: 4 weeks Frequency: 5×/week (30 min)	Control group: Isokinetic training	Sports performance (agility, speed, etc.).	VR group: ↑ Sports performance measures vs. isokinetic training group (*p* < 0.001).
Rusmanto et al. (2023) (37); Indonesia	RCT	Total: 40 junior football athletes Age: 16–20 years Level: Tier 2	Type: Immersive VR football training (shooting + passing) Duration: 12 weeks, 3×/week	Control group: Traditional training	Sports engagement (SES), shooting & passing accuracy	VR group: ↑ Engagement and ↑ technical skills vs. control group (*p* < 0.05).
Shousha et al. (2021) (52); Egypt	RCT	Total: 90 male football players diagnosed with chronic ankle instability Age: 15.2 ± 1.2 years Level: Tier 2	Non-immersive VR (Nintendo Wii Fit Plus). Frequency: 3 sessions per week for 3 months. Duration per session: 60 min (30 min VR + 30 min guideline proprioceptive/balance protocol). Exercises included lunge, single-leg twist, rowing squat, tight-rope walk, and soccer heading tasks.	Biodex balance training group: 30 min Biodex + 30 min guideline protocol Control group: guideline protocol only (60 min).	Functional/neuromuscular outcomes: Cumberland Ankle Instability Tool (CAIT); Overall Stability Index (OASI); Antero-Posterior (APSI); Medio-Lateral (MLSI) indices via Biodex Balance System.	All groups: ↓ OASI, ↓ APSI, ↓ MLSI, and ↑ CAIT (all *p* < 0.001). Between-group: VR and BBT > control for OASI and APSI (*p* < 0.001); no significant difference between VR and BBT (*p* = 1.000).
Wang (2024) (42); China	RCT	Total: 127 university basketball players (65 men, 62 women) Age: 18.2 ± 0.5 years Level: Tier 2	Immersive VR training using VireFit basketball program with VR glasses. Duration: one academic semester (16 weeks). Frequency: regular team sessions including VR component throughout semester. Tasks targeted shooting accuracy, reaction speed, and decision-making under vestibular stimulation.	Traditional basketball training without VR.	Psychophysiological indices (vestibular stability, movement coordination, working memory under vestibular irritation, task switching, etc.). Mental health and psychological mood via Warwick-Edinburgh Mental Well-Being Scale (WEMWBS).	VR group: ↑ Overall psychophysiological scores vs. controls (*p* < 0.001); ↑ mental-health scores (*p* < 0.001); strong correlation between improvements in psychophysiological and mental-health indices (*p* < 0.05). VR training enhanced functional performance and mental well-being more than traditional methods.
Non-randomized controlled studies						
Bedir & Erhan (2021) (49); Turkey	Mixed-method semi-experimental design	Total: 34 elite target-sport athletes (curling = 14, bowling = 13, archery = 7) Age: 21.7 ± 4.3 years Level: Tier 2	Type: Immersive VR-based imagery (VRBI) using 3D GoPro Fusion videos integrated into HMD; included ﻿Physical, Environment, Task, Time, Learn, Emotion, Perspective components and progressive muscle relaxation. Frequency: 3 sessions/week for 4 weeks (2 h each). Content: 3D visual motor rehearsal of sport-specific performance from first-person view.	Visual Motor Behavior Rehearsal + Video Modeling (VMBR + VM) with 2D video imagery, and a passive control group watching sport-related videos.	Primary: Shot performance scores (curling, bowling, archery). Secondary: Movement Imagery Questionnaire–Revised (MIQ-R; visual/kinesthetic imagery). Qualitative: Semi-structured interviews on motivation and concentration.	VRBI group: ↑ shot performance (interaction effect, *p* < 0.001); post-training: VRBI > VMBR + VM > control. ↑ Imagery skill improvements for VRBI and VMBR + VM vs. control (*p* = 0.007). Athletes adapted faster to VRBI with earlier performance gains (week 2). Qualitative data: early stress → ↑ confidence and ↑ focus by week 3.
Gazali et al. (2025) (24); Indonesia	Quasi-experimental study	Total: 56 primary school students Age: 10.7 ± 0.8 years Sport: Badminton Level: Tier 1	Type: Immersive VR Content: VR-based learning of badminton skills (e.g., footwork, stroke mechanics) Duration: 4 weeks	Conventional learning: Traditional badminton training methods.	Engagement and enjoyment, not biomechanical risk markers.	VR group: ↑ Engagement and ↑ enjoyment vs. traditional learning (*p* < 0.05) in badminton, though not a direct injury risk marker.
Gonzalez-Fernandes et al. (2025) (50); Tunisia	Quasi-experimental double-blind trial	Total: 80 elite athletes (40 men, 40 women Age: 18–35 years Sports: football, basketball, swimming, track & field Level: Tier 4	Immersive VR mental-skills training using Meta Quest Pro HMD with sport-specific simulations and real-time biofeedback (HRV, EDA). Duration: 6 weeks. Frequency: 3 × 30-min sessions per week. Adaptive-difficulty algorithm modulated stressors (crowd noise, time pressure) based on HRV coherence.	Traditional mental-skills training (Physical, Environment, Task, Time, Learn, Emotion, Perspective imagery + breathing biofeedback + cognitive restructuring). Same frequency and duration.	Psychological: Sport Mental Toughness Questionnaire (SMTQ); Competitive State Anxiety Inventory-2R (CSAI-2R). Physiological: Heart-rate variability (RMSSD, LF/HF); electrodermal activity (EDA). Performance: Coach-rated decision quality & technical precision; VR analytics (reaction time ms, accuracy %).	VR group: ↑ Mental toughness vs. controls (*p* < 0.001); ↓ cognitive anxiety (*p* < 0.001); ↑ HRV (RMSSD) (*p* < 0.001); ↓ stress recovery time by 40% (*p* = 0.003); ↑ decision accuracy by 23% under pressure (*p* < 0.001). Team-sport athletes benefited more than individuals (*p* = 0.02). Effects retained at 3-month follow-up; minor cybersickness (7.5%) resolved quickly.
Reneker et al. (2020) (21); United states	Quasi-experimental study.	Total: 130 men's and women's soccer teams at two universities Age: 20.5 ± 1.6 Level: Tier 2	Type: Virtual Immersive Sensorimotor Training (VIST) Content: Virtual reality exercises Duration: twice a week for 6 weeks	Historical control was used to compare injury rates	Training effect on VR exercises, balance, and other sensorimotor control measures	VR group: ↑ cervical neuromotor control (*p* < 0.001), ↑ balance (*p* < 0.005), and ↑ inspection time (*p* = 0.008) vs. control group. No significant differences in injury rate or on-field performance metrics between groups (*p* > 0.05).
Trpkovici et al. (2025) (27); Hungary	Comparing VR to a competitive match	Total: 24 female athletes Age: 18.7 ± 5.4 years Sport: Basketball, table tennis, and handball Level: Tier 3	Type: VR effectively simulates competitive stress	Comparator: A competitive match	Psychological responses, anxiety, and stress management skills	VR effectively simulates competitive stress and is a promising tool for SIT to enhance athletes’ stress management skills.
Van Wallendael et al. (2024) (18); Belgium	Investigation of non-immersive XR and immersive VR on movement quality.	Total: 42 male soccer players with a soccer-related ACL tear or healthy Age: 22.9 ± 3.8 years Level: Tier 2	Type: Non-immersive XR and immersive VR Content: Return-to-Sport assessment Duration: Single session	Healthy control players and a comparison between XR and VR	Movement quality during Return-to-Sport assessment post ACL reconstruction.	XR and VR environments: ↑ detection of ACL-related deficits by simulating sport-specific scenarios (*p* < 0.05).
Witte et al. (2022) (41); Germany	Non-randomized controlled trial	Total: 27 athletes (17 males, 10 females) Age: 17.4 ± 3.5 years Sport: Karate Level: Tier 2	Immersive VR (HMD—HTC Vive Pro Eye); 10 min VR + 80 min conventional training per session. Task: respond to a virtual opponent's attacks; progressive difficulty; no haptic feedback. Duration: 10 sessions over 6 weeks	Conventional training only (90 min per session).	Functional/perceptual-cognitive: karate-specific response time (s) and response quality (counterattack count); reaction time tests (Vienna Test System S1/S4); acceptability via simulator sickness questionnaire.	VR group: Significant main effect of time for response time (GZj: *p* < 0.001; KZ: *p* = 0.001); ↑ response time in VR condition only (GZj: *p* < 0.001; KZ: *p* = 0.001). No between-group differences in real-world tests (*p* > 0.05). ↑ Response quality over time (GZj: *p* = 0.028; KZ: *p* = 0.002). ↑ Reaction times (S4) in both groups (*p* < 0.001). No VR-related side effects reported. VR training reduced response time within the VR environment, but transfer to real-world performance was not observed.
Within-subject comparative/repeated measures						
Brazalovich et al. (2024) (34); United states	Within-subjects, repeated measures	Total: 29 physically active adults (22 M, 7 F) Age: 20.52 ± 1.21 years Sport: Drop landing (Military Physical Training) Level: Tier 1	Type: Immersive VR (Head-Mounted Display) Content: A 360circ photo of a steep drop Duration: Single-session assessment	Eyes-Open: Standard lab environment Eyes-Closed: Standard lab environment	Peak vertical ground reaction force, LESS score, knee flexion at initial contact, and knee abduction.	VR condition: ↑ Injury-risk biomechanics during drop landing—↑ vGRF, ↑ LESS errors, and ↑ knee abduction (*p* < 0.05).
Cortes et al. (2011) (40); United states	Within-subjects, repeated measures	Total: 13 female collegiate athletes Age: 19.3 ± 0.9 years Sport: Soccer Level: Tier 3	Type: Non-immersive VR (video simulation) Content: A soccer game situation that cues a random cut or stop Duration: Single-session assessment	Anticipated Condition: No video cue, pre-planned cut	Knee flexion, knee adduction-abduction, and knee rotation during a sidestep cutting task.	VR simulation (unanticipated condition): ↑ Knee adduction and ↑ internal rotation during sidesteps cutting, producing movement patterns linked to higher ACL injury risk (*p* < 0.05).
DiCesare et al. (2019) (35); United states	Within-subjects, repeated measures crossover design	Total: 22 healthy male soccer players Age: 20.4 ± 1.6 years Level: Tier 2	Type: Immersive VR (Head-Mounted Display) Content: Soccer corner kick scenario, where a player heads a ball during a jump-landing task Duration: Single-session assessment.	Standard Condition: A DVJ task in a standard lab setting without VR	Kinematics of the hip, knee, and ankle joints (sagittal and frontal planes) during landing.	VR environment: ↑ Ankle inversion and ↑ hip adduction, producing movement patterns associated with higher ACL injury risk (*p* < 0.05).
Ford et al. (2015) (25); United states	Within-subject comparative randomized crossover	Total: 4 female high school soccer players Age: 14.8 ± 1.0 years Level: Tier 2	Type: Real-time visual biofeedback displayed on a screen Content: Two different modes of biofeedback (kinetic-focused vs. kinematic-focused) during repetitive double-leg squats Duration: Single session, pre- and post-training drop vertical jumps were collected.	The study compared the effects of two different biofeedback modes.	Maximum knee abduction moment and angle during a drop vertical jump.	Biofeedback training: ↓ Knee abduction moment and ↓ knee abduction angle (both *p* < 0.05), indicating improved landing mechanics and reduced high-risk movement patterns.
Harrison et al. (2021) (47); United states	Within-subject, repeated measures	Total: 13 female soccer players Age: 20.5 ± 1.1 years Level: Tier 2	Immersive VR relaxation via Oculus Quest HMD (Liminal VR “campfire” calm scene). Duration: one 4-min session. Frequency: single session after stress induction.	Within-subjects baseline and stress conditions (no VR).	Cognitive and somatic anxiety (MRF-3), self-confidence (MRF-3), mental effort (RSME), heart rate (Polar H10), and kinematics (lumbar & thigh accelerometers during penalty kicks).	VR relaxation: ↓ Cognitive anxiety (*p* < 0.001), ↓ somatic anxiety (*p* < 0.001), and ↑ self-confidence (*p* < 0.001) vs. baseline and stress. ↑ Mental effort under stress vs. baseline (*p* = 0.006). ↓ Heart rate in VR (*p* = 0.02). No significant changes in performance (*p* = 0.21) or kinematics (*p* > 0.05). VR effectively reduced anxiety and HR but did not alter performance or movement mechanics.
Williams et al. (2022) (36); United states	A 3 × 3 repeated-measures design with three groups	Total: 45 healthy adults (21 M, 24 F) Age: 21.9 ± 1.9 years Sport: Not sport-specific Level: Tier 1	Type: Immersive VR (goggles) with biofeedback based on motor learning principles Content: Participants performed single-leg squats while mimicking a VR avatar; received positive biofeedback (green highlights on the avatar) when performing the movement correctly. Duration: Single-day intervention	Control group mimicked the avatar during the single-leg squats but received no additional positive feedback or choices.	Dual-task cost (i.e., the performance decline in balance when adding a simultaneous cognitive task) during a single-leg balance hold, measured by COP velocity.	EE (enhanced expectancy) group: ↑ Motor learning with a 50.6% ↓ in dual-task costs from baseline to retention (*p* < 0.05) vs. control. Autonomy Support (task-irrelevant choice: avatar color) showed no significant advantage over control (*p* > 0.05).
Uncontrolled pre-post/pilot studies						
Diekfuss et al. (2020) (19); United states	Longitudinal investigation (preliminary)	Total: 30 healthy, young, physically active female athletes Age: 14–17 years Level: Tier 2	Group-based ﻿real-time visual feedback NMT Duration: 6 weeks Frequency: 3 sessions per week	﻿Untrained controls	High-risk knee biomechanics (knee valgus), jump-landing kinematics, and functional brain connectivity (via fMRI).	Augmented NMT with biofeedback: ↓ High-risk knee biomechanics (*p* = 0.03) and ↑ functional brain connectivity related to motor control (*p* = 0.04).
Grooms et al. (2018) (39); United states	Pre-post cohort study.	Total: 4 healthy high school female soccer athletes Age: High school age (not specified further). Level: Tier 3	Type: Augmented NMT using real-time visual biofeedback projected onto a screen. Content: A visual rectangle stimulus deformed in real-time based on key injury-risk biomechanics (e.g., knee abduction moment, trunk flexion) during exercises like squats and tuck jumps. The goal was for athletes to implicitly learn to keep the rectangle's shape, which corresponded to low-risk movements. Duration: 6 weeks, 3 times per week	There was no separate comparator group	Landing biomechanics (hip adduction, knee rotation) during a post-training, unanticipated cutting maneuver in a soccer-specific VR scenario to assess transfer of skills. Changes in brain activity were also measured with fMRI.	VR training: ↓ Hip adduction and ↓ knee rotation (*p* < 0.01) during sport-specific tasks; changes in brain activity were strongly correlated with improved, lower-risk landing mechanics.
Kiefer et al. (2017) (26); United states	Pilot study, experimental design	Total: 38 female high school athletes Age: 16.2 ± 1.1 years Sport: Soccer Level: Tier 1	Type: Sport-specific VR biofeedback (non-immersive display) Content: Augmented NMT with biofeedback Duration: 6 weeks, 2–3 times per week	No comparator group, as it was a pilot study to test the intervention's feasibility.	Biomechanical data during an unanticipated cutting task, including knee valgus, and EMG.	VR biofeedback: ↓ Internal hip rotation during unanticipated cutting (*p* = 0.02), indicating effective modification of movement patterns.
Reneker et al. (2019) (22); United states	One-arm experimental pilot	Total: 75 collegiate soccer players (38 male, 37 female) Age: 20.2 ± 1.5 years Level: Tier 2	Type: Sensorimotor training intervention. 8 training sessions over 4 weeks	Historical control: Injury rate was compared to historical data	Clinical measurements of sensorimotor control (e.g., static balance on the Sway app, near-point convergence, etc.).	VR intervention: ↑ Clinical measures of sensorimotor control (*p* < 0.03), indicating improved neuromotor function relevant to injury prevention.

Athlete caliber classified according to the six-tier Participant Classification Framework proposed by McKay et al. (2022): Tier 0 = Sedentary; Tier 1 = Recreationally Active; Tier 2 = Trained/Developmental; Tier 3 = Highly Trained/National Level; Tier 4 = Elite/International Level; Tier 5 = World Class.

ACL, anterior cruciate ligament; COP, center of pressure; DVJ, drop vertical jump; EE, enhanced expectancies; EMG, electromyography; GRF, ground reaction force; HMD, head-mounted display; NMT, neuromuscular training; RCT, randomized controlled trial; SIT, stress inoculation training; VR, virtual reality; VRT, virtual reality training; XR, extended reality; VSS, video-stimulation screen; FAI, functional ankle instability; SEBT, star excursion balance test.

In the terms of methodological design, 13 studies were randomized controlled trial (23, 28, 29, 37, 38, 42–46, 48, 51, 52), seven were non-randomized controlled or quasi-experimental studies (18, 21, 24, 27, 41, 49, 50), six followed a within-subject repeated-measures designs (25, 34–36, 40, 47), and four were uncontrolled pre–post or pilot investigations (19, 22, 26, 53). The majority were conducted in laboratory or sports-specific training settings and evaluated short- to medium-term interventions ranging from single-session experiments to multi-week training programs lasting up to 16 weeks.

The included participants represented a spectrum of athletic levels based on McKay et al., predominantly Tier 1–2 (recreationally active to trained/developmental; *n* = 22), Tier 3 (highly trained/national; *n* = 6), and Tier 4 (elite/international; *n* = 2) (54). This distribution indicates that most available evidence concerns non-elite or developing athletes, whereas evidence involving elite performers remains limited.

Consistent with our review scope, most studies evaluated outcomes relevant to lower-extremity injury risk factors, including knee and ankle biomechanics, landing and cutting mechanics, balance, and postural control. A smaller subset examined perceptual-cognitive or psychological domains considered potentially relevant to lower-extremity injury-risk management, such as decision-making, attentional control, confidence, and stress regulation.

The interventions primarily targeted the modification of biomechanical, neuromuscular, functional and psychological risk factors associated with sports injuries rather than direct reductions in injury incidence. Across studies, commonly assessed outcomes included landing mechanics, joint kinematics, balance and postural stability, reaction time, neuromuscular coordination, perceptual-cognitive function, and psychological responses. A detailed summary of all study characteristics and key findings is presented in Table 1.

### 3.3 Characteristics of the intervention programs

The intervention programs varied considerably in virtual-environment modality, duration, intensity, frequency, and training focus. Interventions were delivered using immersive head-mounted displays, semi-immersive projection or screen-based systems, or non-immersive VR/biofeedback platforms such as Nintendo Wii.

Most interventions were conducted in controlled laboratory or sport-specific training settings, typically with training frequencies ranging from one to three sessions per week. The majority of controlled trials implemented VR- or augmented reality-based neuromuscular and perceptual-cognitive protocols targeting balance, postural control, coordination, movement quality, and decision-making.

Ten studies (18, 25, 27, 28, 34–36, 40, 44, 47) involved acute or very short-term exposure protocol assessing immediate biomechanical or neuromuscular responses, whereas the remaining studies (19, 21, 22–24, 26, 29, 37–39, 41–43, 45, 46, 48–52) evaluated cumulative adaptations following multi-week interventions. Notably, studies employing highly immersive, cognitively demanding, or unfamiliar VR tasks were more likely to report transient increases in potentially higher-risk movement mechanics during early exposure sessions.

### 3.4 Results of the main outcome parameters

#### 3.4.1 Biomechanical risk factors

Twelve studies (18, 19, 25–27, 34, 35, 38–40, 46, 51) evaluated biomechanical outcomes including joint kinematics, landing mechanics, ground-reaction forces, balance indices, and movement asymmetries. Across both acute and multi-week interventions, VR-based protocols generally demonstrated favorable effects on lower-extremity movement patterns commonly associated with injury-risk management, although the direction and magnitude of these adaptations varied according to task design, immersion level, and feedback modality.

Several studies reported improvements in landing mechanics, dynamic balance, lower-limb alignment, and movement coordination following VR-based biofeedback or neuromuscular-training interventions (19, 25, 26, 38, 39, 46, 51). Reported adaptations included reductions in knee valgus, hip internal rotation, movement asymmetries, and postural-instability measures; particularly, during structured multi-week interventions incorporating real-time visual or augmented feedback.

However, not all findings were uniformly favorable. Acute immersive simulations involving unanticipated cutting or landing tasks occasionally produced transient increases in potentially higher-risk lower-extremity mechanics, including greater hip adduction, internal rotation, ankle inversion, or elevated ground-reaction forces (34, 35, 40). These responses were primarily observed during highly immersive, cognitively demanding, unfamiliar, or unanticipated task conditions, suggesting that task novelty, ecological complexity, and early exposure characteristics may substantially influence immediate biomechanical adaptations. In addition, some VR and augmented-reality systems primarily functioned as assessment or diagnostic tools by improving the detection of ACL-related asymmetries or movement deficits rather than directly modifying them (18).

#### 3.4.2 Neuromuscular risk factors

Neuromuscular outcomes related to balance, coordination, proprioception, postural control, and muscle-activation patterns were assessed in 12 studies (19, 21, 22, 23, 36–39, 41, 45, 48, 51). Overall, VR-based interventions generally improved neuromuscular control, dynamic balance, coordination, and reaction efficiency relative to baseline or conventional training conditions.

Reported adaptations included improvements in ankle stability, postural control, movement coordination, reaction-time performance, and motor-learning efficiency (23, 36, 38, 41, 45, 51). Several studies additionally demonstrated favorable changes in neuromuscular activation timing and sensorimotor regulation following VR-based balance or feedback-oriented interventions (19, 22, 39, 51). Although not exclusively focused on the lower extremity in all cases, most interventions targeted movement-control processes relevant to lower-extremity injury-related risk factors.

Studies involving reactive feedback, augmented neuromuscular training, or sport-specific sensorimotor tasks also reported concurrent cortical-activation changes alongside biomechanical or motor-control adaptations (19, 39). Collectively, these findings support the potential of VR-based environments to facilitate short-term neuromuscular and sensorimotor adaptations; however, direct relationships between these changes and verified reductions in injury incidence remain insufficiently established.

#### 3.4.3 Functional and perceptual-cognitive risk factors

Ten studies (18, 24, 28, 29, 37, 42–44, 49, 50) evaluated functional and perceptual-cognitive outcomes including decision-making, attentional control, visual search behavior, coordination, technical execution, and sport-specific task performance.

Overall, VR-based interventions demonstrated improvements in perceptual-cognitive processing, executive attention, reaction efficiency, coordination, and sport-specific decision-making across multiple sports contexts (24, 28, 29, 42–44, 49, 50). Immersive and interactive VR environments were frequently associated with enhanced engagement, skill acquisition, and task-specific performance relative to conventional training approaches.

Several studies further reported improved visual attention, confidence, and movement execution during sport-specific tasks, while VR- and extended-reality systems also enhanced the identification of ACL-related movement deficits and asymmetries (18, 28, 29, 43). These findings suggest that VR-based environments may support perceptual-cognitive adaptations relevant to movement regulation and injury-risk management; however, the extent to which such adaptations directly translate into injury reduction remains uncertain.

#### 3.4.4 Psychological and stress-adaptation risk factors

Five studies (27, 42, 47, 48, 50) examined psychological and stress-adaptation outcomes. Across these studies, VR-based interventions generally improved emotional regulation, stress responses, attentional control, confidence, and mental resilience.

Reported findings included reductions in cognitive anxiety and stress-related responses, improvements in self-confidence and attentional control, and favorable psychophysiological adaptations following immersive VR-based relaxation or mental-skills training protocols (42, 47, 48, 50). Virtual competitive-stress simulations also reproduced psychologically demanding sport environments and elicited realistic emotional and attentional responses (27).

Although these psychological adaptations may indirectly influence movement behavior, sport performance, or training engagement, current evidence remains insufficient to determine whether such responses independently contribute to reductions in sports-injury incidence.

### 3.5 Summary of outcome effects

Across all outcome domains, 28 of the 30 included studies reported improvements in at least one biomechanical, neuromuscular, functional, or psychological outcome following VR-based interventions. The most consistent findings involved improvements in lower-extremity-relevant balance performance, movement coordination, reaction efficiency, perceptual-cognitive processing, and psychological regulation.

Nevertheless, several acute investigations also demonstrated transient increases in potentially higher-risk lower-extremity mechanics, particularly during highly immersive, cognitively demanding, unfamiliar, or unanticipated VR tasks. These findings suggest that although VR-based interventions may facilitate motor learning and sensorimotor adaptation, excessive sensory-cognitive demand, inadequate familiarization, or inappropriate task progression may temporarily increase mechanical loading during early exposure phases.

Importantly, the overwhelming majority of included studies evaluated surrogate biomechanical, neuromuscular, functional, or psychological outcomes rather than verified injury-incidence reductions. Consequently, evidence directly linking these adaptations to actual reductions in sports injuries remains limited.

### 3.6 Quality assessments

Methodological quality, appraised using the Downs and Black checklist, ranged from 14 to 26 across the 30 included studies, with a mean of 19.6 ± 3.1, indicating an overall fair-to-good methodological quality. Fifteen studies (50%) were categorized as good (20–25), thirteen (43%) as fair (15–19), one (3%) as excellent (26–28), and one (3%) as poor (≤14) (Table 2).

**Table 2:** Methodological quality assessment of the included studies using the downs and black checklist (33).

Study	Reporting (10)	External (3)	Bias (7)	Confounding (6)	Power (1)	Total (28)	Quality category
Bedir and Erhan (2021) (49)	10	3	5	4	1	23	Good
Brazalovich et al. (2024) (34)	11	0	5	5	0	21	Good
Cortes et al. (2011) (40)	11	0	5	4	0	20	Good
DiCesare et al. (2019) (35)	9	0	5	4	0	18	Fair
Diekfuss et al. (2020) (19)	9	0	5	3	0	17	Fair
Faghihi and Khanmohammadi (2024) (51)	10	3	6	6	1	26	Excellent
Fendrian et al. (2024) (48)	8	0	5	4	1	18	Fair
Ford et al. (2015) (25)	9	0	5	5	0	19	Fair
Fortes et al. (2021) (29)	9	0	6	5	1	21	Good
Gazali et al. (2025) (24)	10	0	5	5	0	20	Good
Gonzalez-Fernandes et al. (2025) (50)	10	2	7	6	0	25	Good
Grooms et al. (2018) (39)	8	0	5	4	0	17	Fair
Harrison et al. (2021) (47)	10	2	5	4	0	21	Good
Kiefer et al. (2017 (26)	8	0	5	2	0	15	Fair
Marshall et al. (2023) (28)	10	0	5	5	1	21	Good
Mohammadi et al. (2023) (1) (23)	10	0	6	5	1	22	Good
Mohammadi et al. (2023) (2) (45)	10	1	6	6	1	24	Good
Mohamed and EI-Bedewy (2023) (46)	10	3	5	4	0	22	Good
Nambi et al. (2020) (38)	9	0	7	4	1	21	Good
Novak et al. (2023) (44)	7	1	5	4	1	18	Fair
Rahmadani et al. (2024) (43)	9	1	5	4	1	20	Good
Reneker et al. (2019) (22)	8	0	5	1	0	14	Poor
Reneker et al. (2020 (21)	9	0	5	2	0	16	Fair
Rusmanto et al. (2023) (37)	8	0	5	4	0	17	Fair
Shousha et al. (2021) (52)	10	3	5	6	1	25	Good
Trpkovici et al. (2025) (27)	8	0	5	3	0	16	Fair
Van Wallendael et al. (2024) (18)	9	0	5	3	0	17	Fair
Williams et al. (2022) (36)	10	0	5	5	0	20	Good
Wang (2024) (42)	8	1	5	4	0	18	Fair
Witte et al. (2022) (41)	8	0	5	3	0	16	Fair

Scores reflect the number of items satisfied in each domain of the Downs and Black checklist (maximum = 28).

Quality categories: Excellent (26–28); Good (20–25); Fair (15–19); Poor (≤14).

Randomized controlled trials generally demonstrated higher methodological quality than non-randomized or uncontrolled investigations, reflecting clearer reporting, more structured intervention procedures, and more appropriate statistical analyses. However, important methodological limitations remained common across study designs, including limited blinding procedures, small sample sizes, absence of formal power calculations, and restricted long-term follow-up.

Methodological rigor appeared to vary across outcome domains, with studies evaluating biomechanical and neuromuscular outcomes more frequently employing controlled designs and objective outcome measures, whereas studies focusing on perceptual-cognitive or psychological outcomes often relied on smaller samples or more exploratory methodologies. Across all study designs, reporting quality represented the strongest methodological domain, whereas external validity and statistical power were the weakest. Only 11 studies (36.7%) reported a formal *a priori* sample-size or power calculation.

## 4 Discussion

The main finding of this systematic review is that VR-based interventions consistently modified biomechanical, neuromuscular, functional, and psychological outcomes that may be relevant to lower-extremity injury-related risk factors across 30 included studies. Most interventions improved balance, motor control, movement coordination, decision-making, or emotional regulation, although few directly examined injury-incidence outcomes. Importantly, the included interventions primarily targeted surrogate risk-related parameters rather than demonstrating confirmed reductions in actual sports injuries. Overall, VR appears to represent a feasible and engaging approach for retraining lower-limb movement quality and perceptual–motor coordination while potentially supporting psychological readiness and training engagement.

Several studies demonstrated that VR-based or augmented neuromuscular training modified lower-limb biomechanics commonly associated with injury-risk management. Ford et al., Kiefer et al., Diekfuss et al., and Faghihi & Khanmohammadi observed reductions in knee valgus, altered movement mechanics, or improved neuromuscular timing following VR-enhanced training (19, 25, 26, 51), findings generally comparable to traditional neuromuscular training programs (8, 55). Such adaptations may reflect improved proprioceptive awareness and sensorimotor reorganization facilitated by real-time visual feedback in immersive environments.

In contrast, several acute investigations (34, 35, 40) reported transient increases in potentially higher-risk mechanics—including greater hip adduction, internal rotation, ankle inversion, or elevated ground-reaction forces—particularly during highly immersive, unfamiliar, or cognitively demanding VR tasks. These findings may reflect temporary motor-control disturbances associated with ecological novelty, delayed sensorimotor adaptation, excessive attentional demand, or altered movement strategies during early exposure phases (56, 57). Consequently, VR environments may simultaneously provide corrective neuromuscular feedback while also imposing elevated sensory-cognitive demands depending on task complexity, immersion level, exposure duration, and user familiarity.

In the neuromuscular domain, consistent improvements were reported in lower-limb balance, postural stability, and coordination, with several studies reporting improvements comparable to, or in some cases greater than, those observed following conventional training approaches; however, substantial methodological heterogeneity limits definitive comparative conclusions. Mohammadi et al. (23) and Nambi et al. (38) found superior gains in dynamic balance and agility, while Reneker et al. (21, 22) demonstrated enhanced sensorimotor control and reaction time through virtual environments. Williams et al. (36) and Witte et al. (41) reported that expectancy-based or reactive feedback reduced dual-task costs, indicating greater neuromuscular efficiency. These short- to mid-term improvements reflect effective neural and motor adaptation; however, the persistence of these effects beyond the intervention period remains uncertain due to limited longitudinal follow-up.

Evidence also supports the functional and perceptual–cognitive benefits of VR training. Fortes et al. (29) and Marshall et al. (28) showed that immersive VR improved decision-making accuracy, visual search, and heading performance compared with conventional practice. Rahmadani et al. (43), Novak et al. (44), and Wang (42) reported significant improvements in executive attention, reaction speed, and coordination, confirming that VR tasks can enhance perceptual–cognitive integration relevant to sport. These results parallel prior evidence suggesting that faster visual anticipation and decision-making may contribute to more effective movement regulation during sport-specific tasks associated with non-contact lower-extremity injury mechanisms (53, 58). Nonetheless, a few studies observed elevated mechanical demands under complex immersive conditions, suggesting that excessive cognitive or motor load may momentarily increase injury risk if progression is not individualized.

From a psychological perspective, VR environments appear capable of influencing emotional regulation, attentional control, confidence, and stress responses during sport-related tasks. Harrison et al. (47) and Trpkovici et al. (27) demonstrated reductions in anxiety and enhanced attentional focus during immersive competitive simulations, while Gonzalez-Fernandes et al. (50), Fendrian et al. (48), and Wang (42) reported improvements in mental resilience, attentional regulation, and psychophysiological responses. Nevertheless, the contribution of these psychological adaptations to actual injury reduction remains speculative. Although psychological readiness, attentional allocation, and stress regulation may influence movement behavior and decision-making under pressure, current evidence does not establish a direct causal relationship between these outcomes and reduced sports-injury incidence (59, 60). Rather, such factors may function as indirect or mediating influences within the broader multifactorial context of injury risk, requiring more research to clarify.

An additional consideration emerging from the present findings is the potential for mechanical overload and attentional fatigue during highly immersive or cognitively demanding VR exposure. Several acute investigations demonstrated transient increases in ground-reaction forces, altered cutting mechanics, or less favorable lower-extremity kinematics during unfamiliar or unanticipated VR tasks (34, 35, 40). One possible explanation is that immersive environments may initially impose elevated sensory-cognitive demands that temporarily exceed an athlete's adaptive motor-control capacity; particularly, when rapid visuomotor integration, dual-task processing, or reactive decision-making are simultaneously required. Such conditions may promote compensatory movement strategies, delayed neuromuscular responses, or altered landing mechanics before adequate familiarization and motor adaptation occur.

Similarly, prolonged or cognitively intensive VR exposure may contribute to attentional fatigue, characterized by reduced attentional allocation, delayed perceptual processing, and diminished executive-control efficiency. Previous cognitive-motor literature suggests that excessive sensory load, immersive complexity, or sustained divided-attention demands may impair motor performance and increase movement variability, particularly under fatigue or time-pressure conditions (57, 61). Consequently, gradual progression, task familiarization, individualized load calibration, and careful monitoring of cognitive demand may represent important considerations when implementing VR-based training within injury-risk-management contexts.

Beyond mental preparation, VR provides a controlled and feedback-rich platform for physical technique refinement. Motion-capture and biofeedback technologies allow athletes to receive real-time feedback on lower-limb joint kinematics and landing posture, promoting precise technique adjustments and efficient motor execution. By reproducing complex sport-specific conditions in a safe digital environment, VR may support motor-learning transfer and movement-pattern rehearsal within controlled environments while potentially reducing exposure to uncontrolled real-world training conditions.

The playing level of participants varied across studies, with most involving Tier 1–2 (recreationally active or trained/developmental) athletes, a smaller proportion of Tier 3 (highly trained/national), and only two studies including Tier 4 (elite/international) performers. This distribution suggests that current evidence mainly reflects youth and sub-elite populations, who may benefit most from VR's feedback-driven learning mechanisms. Elite athletes, conversely, might exhibit smaller relative improvements due to established neuromuscular efficiency, highlighting the need for tier-specific investigations and more research at an elite level.

Few systematic reviews have addressed VR or extended reality interventions in sports injury prevention. Schuermans et al. (14) and Soltanabadi et al. (17) primarily examined rehabilitation and ACL recovery, while Demeco et al. (15) summarized VR in football with a focus on post-injury recovery. In contrast, the present review uniquely synthesizes preventive and performance-oriented evidence across multiple sports, emphasizing biomechanical, neuromuscular, and psychological risk factors of lower limbs rather than clinical rehabilitation outcomes. The inclusion of perceptual-cognitive and mental-training studies broadens the understanding of how VR can holistically address both physical and psychological determinants of lower-extremity injury risk.

### 4.1 Methodological considerations and limitations

Despite promising findings, several limitations remain. Most included studies examined short-term adaptations in surrogate lower-extremity risk factors rather than verified injury-incidence outcomes, representing a key methodological gap. Furthermore, many of these biomechanical, neuromuscular, functional, and psychological outcomes have not been conclusively validated as independent predictors of future sports injuries. Consequently, improvements in these variables should not be interpreted as direct evidence of injury prevention or injury-rate reduction. Heterogeneity in VR modality, intervention duration, frequency, and outcome metrics limited quantitative synthesis. Only 11 studies (36.7%) conducted *a priori* power analyses, and few implemented assessors blinding or randomization concealment. Sample sizes were small, interventions short-term, and follow-up assessments rare, restricting conclusions on durability and transfer to on-field injury prevention. Variations in control conditions (traditional training, no intervention, or alternate digital methods) may also affect comparisons. Although the majority of findings were positive, the potential for transient increases in injury risk during immersive VR exposure should not be ignored. Appropriate safety monitoring, progressive adaptation, and familiarization phases are therefore essential to mitigate this risk.

Potential publication and language biases should also be considered. Because only English-language studies were included, relevant evidence published in other languages may have been overlooked. In addition, emerging technology fields such as VR may be particularly vulnerable to publication bias, novelty effects, and expectancy-driven responses, whereby positive or innovative findings are more likely to be published and participant engagement may be temporarily enhanced due to the immersive or unfamiliar nature of the intervention itself. These factors may partially contribute to the predominance of favorable short-term findings reported across the current VR literature.

### 4.2 Practical implications and future directions

The findings have practical implications for sports scientists, coaches, and clinicians. VR-based programs can complement conventional injury-risk-management strategies by offering interactive and ecologically enriched simulations capable of reproducing sport-specific cognitive and lower-limb mechanical demands. Integration with neuromuscular and perceptual-cognitive exercises may support engagement, motor learning, and feedback precision; however, these applications should currently be interpreted as exploratory and hypothesis-generating rather than definitive preventive solutions. Current evidence supports the use of VR as a tool for modifying injury-related risk factors rather than as a proven injury-prevention intervention. Emerging mixed-reality and extended-reality systems integrating wearable motion sensors, real-time biomechanical feedback, motion-capture technology, and adaptive task environments may further enhance individualized movement assessment and training specificity in future applications.

## 5 Conclusion

In conclusion, VR-based interventions elicit measurable changes in biomechanical, neuromuscular, perceptual-cognitive, and psychological domains associated with lower-extremity injury-risk management. While evidence supports modification of several surrogate lower-limb risk-related factors, direct confirmation of injury-rate reduction and long-term safety remains limited. Findings across 30 studies suggests that VR may represent a promising adjunct to conventional neuromuscular and perceptual-cognitive training approaches; however, current evidence remains insufficient to confirm definitive preventive efficacy at the injury-incidence level. Careful progression, familiarization, safety oversight, and individualized task calibration appear particularly important to minimize excessive mechanical loading, sensory-cognitive overload, or attentional fatigue during immersive exposure. Current research remains predominantly limited to recreationally active, developmental, youth and sub-elite athletes, with comparatively little evidence involving elite or international-level performance. Consequently, the generalizability of these findings to high-performance sporting populations remains uncertain. Furthermore, available evidence remains largely focused on lower-extremity interventions, with comparatively little research addressing upper-extremity applications. Future high-quality randomized trials incorporating larger samples, longer follow-up periods, standardized protocols, and verified injury-incidence outcomes are required to determine the real-world preventive value, safety, and clinical applicability of VR-based interventions in sports settings.

## Data Availability

The original contributions presented in the study are included in the article/Supplementary Material, further inquiries can be directed to the corresponding author.
