# Supplementary material for: Virtual reality-based interventions targeting sports injury-related risk factors: a systematic review of biomechanical, neuromuscular, and functional outcomes
Source: Front Sports Act Living. 2026 Jun 30;8:1744369. doi: 10.3389/fspor.2026.1744369 (PMC13367076; doi:10.3389/fspor.2026.1744369)
Supplement: Supplementary file 1 [file Table1.docx]

Supplementary Material

**Supplementary Table 1.** Search string for each database

| Database | Search Strategy |
| --- | --- |
| PubMed/MEDLINE | ( "Virtual Reality"[Mesh] OR "Augmented Reality"[Mesh] OR "Virtual Reality"[tiab] OR "Augmented Reality"[tiab] OR "Extended Reality"[tiab] OR Exergam*[tiab] OR VR[tiab] OR AR[tiab] OR XR[tiab] ) AND ( "Athletic Injuries"[Mesh] OR "Sports Injuries"[Mesh] OR "injury prevention"[tiab] OR "injury risk"[tiab] OR "sports injury"[tiab] OR "athletic injury"[tiab] ) AND ( "Athletes"[Mesh] OR athlete*[tiab] OR sport*[tiab] OR player*[tiab] ) |
| Scopus | ( TITLE-ABS-KEY ( "virtual reality" OR "augmented reality" OR "extended reality" OR exergam* OR vr OR ar OR xr ) ) AND ( TITLE-ABS-KEY ( "injury prevention" OR "injury risk" OR "sports injury" OR "athletic injury" ) ) AND ( TITLE-ABS-KEY ( athlete* OR sport* OR player* ) ) |
| Web of Science | TS=(("virtual reality" OR "augmented reality" OR "extended reality" OR exergam* OR vr OR ar OR xr)) AND TS=(("injury prevention" OR "injury risk" OR "sports injury" OR "athletic injury")) AND TS=((athlete* OR sport* OR player*)) |
| SportDiscus (EBSCOhost) | ( TI ( "virtual reality" OR "augmented reality" OR "extended reality" OR exergam* OR VR OR AR OR XR ) OR AB ( "virtual reality" OR "augmented reality" OR "extended reality" OR exergam* OR VR OR AR OR XR ) ) AND ( TI ( "injury prevention" OR "injury risk" OR "sports injury" OR "athletic injury" ) OR AB ( "injury prevention" OR "injury risk" OR "sports injury" OR "athletic injury" ) ) AND ( TI ( athlete* OR sport* OR player* ) OR AB ( athlete* OR sport* OR player* ) ) |

**Supplementary Table 2.** Excluded studies with brief reasons

| **Study** | **Reason for Exclusion** |
| --- | --- |
| Aydogdu & Sarı, 2018 | Rehabilitation only |
| Baltaci et al. 2012 | Rehabilitation only |
| Baltacı et al. 2013 | Rehabilitation only |
| Barrios et al. 2010 | Non-athlete sample |
| Bonnette et al. 2020 | Non-empirical |
| Burcal et al. 2021 | Non-empirical |
| Condino et al. 2019 | Non-athlete sample |
| ﻿Demeco et al. 2024 | ﻿Narrative review |
| Düking et al. 2018 | Non-empirical |
| Gérin-Lajoie et al. 2010 | Rehabilitation only |
| Gianola et al. 2020 | Non-active sample |
| Gokeler et al. 2013 | Rehabilitation only |
| Gokeler et al. 2014 | Rehabilitation only |
| Gokeler et al. 2013 | Rehabilitation only |
| Gokeler et al. 2016 | Rehabilitation only |
| Ibrahim et al. 2016 | Non-active sample |
| Karakoc et al. 2016 | Rehabilitation only |
| Nambi et al. 2020 | Rehabilitation only |
| Noehren et al. 2011 | Rehabilitation only |
| Punt et al. 2015 | Rehabilitation only |
| Punt et al. 2017 | Rehabilitation only |
| Schuermans et al. 2022 | Review article |
| Soltanabadi et al. 2023 | Scoping review |
